# St John's Wort (*Hypericum perforatum L.*) Photomedicine: Hypericin-Photodynamic Therapy Induces Metastatic Melanoma Cell Death

**DOI:** 10.1371/journal.pone.0103762

**Published:** 2014-07-30

**Authors:** Britta Kleemann, Benjamin Loos, Thomas J. Scriba, Dirk Lang, Lester M. Davids

**Affiliations:** 1 Redox Laboratory and Confocal and Light Microscope Imaging Facility, Department of Human Biology, Faculty of Health Sciences, University of Cape Town, Cape Town, South Africa; 2 Department of Physiological Sciences, Stellenbosch University, Stellenbosch, South Africa; 3 South African TB Vaccine Initiative, Institute of Infectious Disease and Molecular Medicine and School of Child and Adolescent Health, University of Cape Town, Cape Town, South Africa; University of Tennessee, United States of America

## Abstract

Hypericin, an extract from St John's Wort (*Hypericum perforatum L.*), is a promising photosensitizer in the context of clinical photodynamic therapy due to its excellent photosensitizing properties and tumoritropic characteristics. Hypericin-PDT induced cytotoxicity elicits tumor cell death by various mechanisms including apoptosis, necrosis and autophagy-related cell death. However, limited reports on the efficacy of this photomedicine for the treatment of melanoma have been published. Melanoma is a highly aggressive tumor due to its metastasizing potential and resistance to conventional cancer therapies. The aim of this study was to investigate the response mechanisms of melanoma cells to hypericin-PDT in an *in vitro* tissue culture model. Hypericin was taken up by all melanoma cells and partially co-localized to the endoplasmic reticulum, mitochondria, lysosomes and melanosomes, but not the nucleus. Light activation of hypericin induced a rapid, extensive modification of the tubular mitochondrial network into a beaded appearance, loss of structural details of the endoplasmic reticulum and concomitant loss of hypericin co-localization. Surprisingly the opposite was found for lysosomal-related organelles, suggesting that the melanoma cells may be using these intracellular organelles for hypericin-PDT resistance. In line with this speculation we found an increase in cellular granularity, suggesting an increase in pigmentation levels in response to hypericin-PDT. Pigmentation in melanoma is related to a melanocyte-specific organelle, the melanosome, which has recently been implicated in drug trapping, chemotherapy and hypericin-PDT resistance. However, hypericin-PDT was effective in killing both unpigmented (A375 and 501mel) and pigmented (UCT Mel-1) melanoma cells by specific mechanisms involving the externalization of phosphatidylserines, cell shrinkage and loss of cell membrane integrity. In addition, this treatment resulted in extrinsic (A375) and intrinsic (UCT Mel-1) caspase-dependent apoptotic modes of cell death, as well as a caspase-independent apoptotic mode that did not involve apoptosis-inducing factor (501 mel). Further research is needed to shed more light on these mechanisms.

## Introduction

Dismally, metastatic melanoma remains a death sentence. Despite numerous advances molecularly and therapeutically [1]–[4], the death resistance displayed by these cancer cells remains an aspect to be addressed. Clinically, the gold standard remains early detection, surgical resection, followed by bouts of chemo-or radiation therapy [5]. Unfortunately, traditional chemo- and radiation therapy have also been reported to evoke resistance [2], [6]. Moreover, the incidences of melanoma skin cancer continue to rise with the current status at 132,000 melanoma skin cancers occurring globally each year (World Health Organization http://www.who.int/uv/faq/skincancer/en/index1.html) [7]. A number of factors have been implicated in contributing to the heterogeneity of this cancer including both nature and nurture effects [8]. Biologically, these factors seem to be related to specific mutations, cell death evading mechanisms, cellular transporters and the absence or presence of the ultraviolet (UV) light-absorbing pigment, melanin which has been shown to chelate therapeutic agents and produce an hypoxic environment due to increased oxygen consumption [9], [10]. Moreover, Slominski et al, (2009) argue that these features could affect the efficacy of chemotherapy, radiotherapy or photodynamic therapy [11]. Logically therefore a therapeutic intervention should address these issues.

The use of photodynamic therapy (PDT) as an anti-cancer therapy has gained momentum over the past decade with a number of reports revealing its efficacy with respect to bladder, oesophageal, glioblastoma and non-melanoma skin cancers [12]. Further evidence of its efficacy in solid lungadenocarcinoma A549 tumors in nude mice was highlighted by Jakubowska et al. (2013) who showed that the level of nitrosylhemoglobin increases in the course of PDT leading to decreased tumor size [13]. More recently, our group and others have shown its high potential as a therapeutic option in the fight against melanoma skin cancer [14]–[24]. PDT for cancer involves 2 stages. The photosensitizer is first administered topically or intravenously, followed by irradiation of the tumour site with light of a specific wavelength [12]. Following cellular uptake of the photosensitizer, its activation by this light produces reactive oxygen species (ROS) in the presence of molecular oxygen. These ROS have short half-lives and small radii of diffusion and thus exert their action in the vicinity of their production [25], [26]. Accordingly, the intracellular localization of a photosensitizer directly impacts its cytotoxic action [27]. PDT-induced cytotoxicity has been shown to elicit tumor cell death by various mechanisms including apoptosis, necrosis and autophagy-related cell death [27]–[29]. Interestingly, melanomas display a basal level of autophagy that has been recognized by pathologists for many years now. The presence of autophagy-associated organelles (autophagosomes) and melanized melanosomes have previously been reported on [30]–[32]. In addition, it has been proposed that the presence of autophagy in malignant melanoma is consistent with findings that these cells are under constant endoplasmic reticulum (ER) stress, a known inducer of autophagy [33], [34] and effective treatment proposals have therefore included anti-autophagic regimes [35].

As the photosensitizer used in PDT forms part of the armamentarium, it is imperative that its characteristics determine the efficacy within the tumorigenic site or metastatic cells. Hypericin, an extract from St John's Wort (*Hypericum perforatum L.*), is a promising photosensitizer in the context of clinical PDT due to its excellent photosensitizing properties and tumoritropic characteristics [36]–[38]. Hypericin has been touted as one example of a multi-targeting molecule which can inhibit angiogenesis [39] and the development of further metastases [40] through its effect on several key intracellular pathways [41]. An added advantage is that mutations that result in chemo- and radioresistant tumours do not overly affect the sensitivity to PDT due to its unique cytotoxicity mechanisms, underlining the potential of this treatment for melanoma [12].

With this in mind, this study investigated the response mechanisms of pigmented and unpigmented melanoma cells to hypericin-PDT in an *in vitro* human culture model. We present data that shows hypericin uptake and its specific association with intracellular organelles in melanoma cells. Moreover, melanoma cell death mechanisms are elucidated in response to the killing-dose of light-activated hypericin. Overall, this study demonstrates the effectiveness of hypericin-PDT in killing both pigmented and unpigmented melanoma cells by the induction of apoptosis.

## Materials and Methods

### Cell culture

A375 melanoma cells were purchased from the American Type Culture Collection (ATCC, CRL-1619). The cells were originally obtained from a malignant melanoma of the skin of a 54 year old female patient and described as unpigmented, exhibiting an epithelial morphology, adherent growth property, hypotriploid with a modal number of 62 chromosomes, 9 marker chromosomes that are commonly found in each cell and normal N2, N6 and N22 are present at one copy per cell and growing in immunocompromised mice. The 501mel cells were a gift from Prof. Sharon Prince, Dept Human Biology, University of Cape Town, originally derived from lymph node metastases, the gender and age of the patient is not known [42]. The UCT Mel-1 melanoma cells were a gift from Dr EL Wilson, Dept of Haematology, Groote Schuur Hospital, Cape Town. These cells originated from a 67 year old female patient with the primary tumour on the right ankle and the secondary tumour resulting in the cell line from the right inguinal lymph node. The cells were described as exhibiting an epithelioid morphology, pigmented in biopsy, triangular dentritic morphology, a modal chromosome number of 74 and growing in soft agar and nude mice [43]. All cell lines were cultured in Dulbecco's Modified Eagle's Medium (DMEM, Highveld Biological, No. P02, Johannesburg South Africa) supplemented with 10% (v/v) heat inactivated fetal bovine serum (Highveld Biological, No. 306, Johannesburg South Africa) and 100 U/ml penicillin/100 µg/ml streptomycin (Sigma, P3032/S91370, St. Louis, MO, USA) and grown at 37°C, 5% CO_2_. All cells were routinely checked for mycoplasma contamination with the Hoechst 33342 nuclear stain or with a MycoAlert mycoplasma detection kit (Lonza, LT07-318, Basel, Switzerland), according to the manufacturer's instructions. Cells were cultured to 90% confluence for all experiments to obtain similar pigmentation levels.

### Hypericin-PDT

Hypericin, derived from *Hypericum perforatum L.* was obtained from Sigma (56690, St. Louis, MO, USA). A 2 mM stock solution in dimethyl sulfoxide (Merck, 8.02912.1000, Darmstadt, Germany) was stored as aliquots at −80°C. Working solutions were prepared fresh at a concentration of 50 µM in phosphate-buffered saline (1× PBS) and then further diluted for experiments in complete cell culture medium containing 10% (v/v) fetal bovine serum. Hypericin was activated with 1 J/cm^2^ UVA, using a light box with two F15T8 15W/UVA PUVA lamps (320–410 nm, peak 351 nm, Waldmann, 451415530, Villingen-Schwenningen, Germany). The power output was measured using a portable UV meter (Waldmann, Type 585100, Villingen-Schwenningen, Germany). Time of irradiation was calculated with the following equation: Time = Light dose (J/cm^2^)/Irradiance (W/cm^2^) and equated to 6 minutes, 10 seconds. The cells were covered with lids and irradiated from above in 1× PBS. Controls were sham-irradiated by covering the dish with foil. Melanoma cells were exposed to 4 hours of hypericin in complete media to maximize uptake, followed by light activation. At different time points after treatment, the cells were harvested for various analyses. For each experiment cells were exposed to 4 different treatments:

vehicle with sham-irradiation (control −light),
vehicle with light-activation (control +light),
hypericin with sham-irradiation (hypericin −light)
hypericin with light-activation (hypericin +light).

All experiments were carried out under subdued light conditions. The same tissue culture consumables were used for all experiments (Greiner Bio-One, Frickenhausen, Germany), to ensure no differences in light activation.

### Tyrosinase assay

Melanin biosynthesis can be initiated from either the hydroxylation of L-phenylalanine to L-tyrosine or directly from L-tyrosine, which is then hydroxylated to L-dihydroxyphenylalanine (L-DOPA), an obligatory step both in vitro and in vivo. L-DOPA serves as a precursor to both melanins and catecholamines, acting along separate pathways. The copper-containing enzyme, tyrosinase [EC 1.14.18.1], catalyzes three distinct reactions in the melanogenic pathway: hydroxylation of monophenol (L-tyrosine), dehydrogenation of catechol (L-DOPA), and dehydrogenation of DHI; L-DOPA serves as cofactor in the first and third reactions [44]. These reactions involve oxygen uptake [45]. The next step of the pathway is the oxidation of L-DOPA to dopaquinone, a step which is common to both the eu- and pheomelanogenic pathways. Eumelanogenesis involves the further transformation of dopaquinone to leukodopachrome, followed by a series of oxidoreduction reactions with production of the intermediates dihydroxyindole (DHI) and DHI carboxylic acid (DHICA), that undergo polymerization to form eumelanin [9]. Utilization of radiolabelled ^14^C tyrosine with the addition of DOPA enables the assessment of the enzymatic activity of tyrosinase from total cell extracts by detection of the radiolabelled reaction products [46], [47]. In brief, equal cell numbers were cultured to confluency in 6 cm tissue culture dishes for 3 days followed by trypsinisation with trypsin-EDTA (0.05% T/0.02% E, Sigma, T4799/E9884, St. Louis, MO, USA). After centrifugation the cell pellet was washed with 1× PBS and transferred to a 1.5 ml microfuge tube on ice. The PBS wash procedure was repeated to remove all traces of medium, after which the pellet was re-suspended in 1 ml of 0.1 M sodium phosphate buffer (pH 7.2). The samples and their appropriate controls were repeatedly freeze-thawed in liquid nitrogen and then centrifuged at 12000 rpm at 4°C for 20 min. The supernatant was transferred to clean 1.5 ml microfuge tubes and stored at −80°C till use.

The sample protein concentration was determined using the BCA Protein Assay Kit according to the manufacturer's instructions (Thermo Scientific Pierce, 23225, Waltham, Massachusetts, USA) and 120 µg/ml was used for the assay. The cell extract was combined with a solution of L-[U-^14^C]Tyrosine (Amersham, 20 µCi/ml, 1.85 MBq/ml, CFB74, Amersham, UK) and 0.25 mM DOPA (Sigma, D9628, St. Louis, MO, USA) at 37°C for 1 hour. Samples were transferred to 25 mm diameter glass microfiber filters (Whatman, 1822-025, GE Healthcare Life Sciences, Little Chalfont, United Kingdom) and left to air-dry followed by a series of washes in 0.1 N HCl (Merck, SAAR3063054LCA, Darmstadt, Germany), 95% ethanol (Merck, 1.00983.2500, Darmstadt, Germany) and a final wash in acetone (Merck, SAAR1022020LC, Darmstadt, Germany). The discs were then placed in scintillation vials (Sigma, St. Louis, MO, USA) and left to air-dry overnight. The following day, 5 ml scintillation fluid (Zinsser Analytic, Quicksafe A scintillation cocktail, 1008000, Frankfurt, Germany) was added per vial and the samples were read on a Tri-Carb 2100 TR liquid scintillation analyser (Packard, USA). Results were expressed as counts per minute (cpm) of incorporated radioactive tyrosine.

### Hypericin uptake assay

Similar to a previous protocol [48], 3×10^4^ cells were seeded in 35 mm tissue culture dishes overnight. Cells were then exposed to 4 hours of hypericin in complete cell culture medium containing 10% (v/v) fetal bovine serum. All the media was then removed and cells washed with ice-cold 1× PBS. Complete extraction buffer (40 µl, 100 µM Tris HCL, 1% Nonidet P-40, 0.01% SDS, 0.001 mg/ml Aprotinin and 0.1 mM PMSF, Sigma, St. Louis, MO, USA) was added to the dish and cell lysates collected using a rubber syringe stopper. Cell lysates were then centrifuged at 12000 rpm for 20 min at 4°C. The supernatants were divided for the determination of protein content and for the quantification of hypericin fluorescence on a fluorimeter (Cary Eclipse Fluorimeter, Varian, Palo Alto, CA, USA). Hypericin was excited with 563 nm and emission was recorded at 608 nm. Protein concentrations were determined using the BCA assay according to the manufacturer's instructions (Thermo Scientific Pierce, BCA protein assay kit, 23225, Waltham, Massachusetts, USA). Resulting data was normalized to total protein and presented as arbitrary fluorescent units (AFU) per microgram of protein.

### Confocal fluorescent microscopy

Several organelle-specific green and yellow fluorescent protein (G/YFP) plasmids or probes were used to label intracellular organelles of melanoma cells (Table 1). All constructs used in this study were prepared according to standard techniques [49].

**Table 1:** Organelle-specific G/YFP-fusion plasmids and dyes used to visualize intracellular organelles.

Intracellular organelle	Plasmid/Dye	Experimental conditions	Function	Source
Endoplasmic reticulum	pGFP-Bcl2CB5	200 ng of plasmid	Anti-apoptotic ER targeted BCL2 family member	Addgene plasmid 18000 [116]
Endoplasmic reticulum	pEYFP-ER	200 ng of plasmid	Calreticulin, multifunctional quality control of protein folding	Dr G. Schafer, Dr C. Kaschula, ICGEB, University of Cape Town, Clontech 6906
Mitochondria	pEGFP-pOTC	200 ng of plasmid	Ornithine transcarbamylase (OTC), mitochondrial matrix protein	Dr L.M. Davids, University of Cape Town [117]
Lysosomes, late endosomes, melanosomes	LysoTracker Yellow-HCK-123	75 nM for 30 min in medium, live	Acidotropic probe labeling acidic organelles	Invitrogen Molecular Probes, L12491
Lysosomes, endosomes and melanosomes	pYFP-LAMP1	500 ng of plasmid	Lysosomal-associated membrane protein 1 (LAMP1), membrane glycoprotein, provides selectins with carbohydrate ligands	Prof. A.M. Cuervo, NY, Einstein College, USA [118]
Early and late endosomes, lysosomes, early and intermediate stage melanosomes	pEGFP-rab7WT	100 ng of plasmid	Microtubule-based transport, facilitates transport of tyrosinase and TYRP1 from trans-golgi to melanosomes	Addgene plasmid 12605 [119]
Mature melanosomes	pGFP-Rab27a	200 ng of plasmid	Localises to the mature melanosomal membrane, acts as a receptor for myosinVa in the actin-dependent transport of mature melanosomes	Prof. J. Lambert, University of Ghent [120]
Mature melanosomes	pGFP-MyosinVa	200 ng of plasmid	Actin-dependent motor protein, links mature melanosomes to the actin network	Prof J. Lambert, University of Ghent [121]
Nuclei	Hoechst 33342	1 µg/ml for 20 min in medium, live	Binds to DNA	Invitrogen Molecular Probes, H1399

A summary of the experimental conditions, functions and sources of the organelle-specific G/YFP-fusion plasmids and dyes used in this study.

Cells were seeded onto 35 mm^2^ microscope cover glasses at a density of 2×10^4^ cells and allowed to adhere and grow over 48 hours. Cells were then transiently transfected with the plasmids (Table 1). TransFectin (1 µl, Biorad, 170-3351, Hercules, CA, USA) was used for transient transfection of A375 and 501mel and GeneCellin (0.5 µl, BioCellChallenge,GC500, Toulon, France) was used for UCT Mel-1; according to the specific manufacturer's instructions. Controls of the parental GFP/YFP plasmid were included.

At 20 hours after transfection the cells were exposed to 3 µM hypericin for 4 hours. Nuclei were visualized using Hoechst (1 µg/ml, Table 1, Invitrogen Molecular Probes, Carlsbad, CA, USA). To stain lysosomes, 7.5 µl of 10 mM lysotracker yellow (75 nM, Table 1, Invitrogen Molecular Probes, Carlsbad, CA, USA) was added 30 minutes before the end of the hypericin incubation. At the end of the staining period the cells were viewed live, using a confocal fluorescent microscope. Both the Carl Zeiss 510 LSM meta with NLO confocal microscope (LSM and ZEN 2009 software, Oberkochen, Germany) and the Carl Zeiss LSM 780 confocal microscope (ZEN 2009, 2011 software, Oberkochen, Germany) were used in this study, with the Plan-Apochromat 63×/1.4 oil immersion DIC M27 lens.

Fluorophores were excited at the following excitation wavelengths: GFP 488 nm, YFP 514 nm, Lysotracker 458 nm, Hoechst 405 nm, Hypericin 561 nm and emission were collected using the following bandwidths: GFP 490–543 nm, YFP 516–586 nm, Lysotracker 464–604 nm, Hoechst 410–516 nm, Hypericin 585–734 nm. Profiles taken through different areas of the cell displaying the fluorophores were created using free Zen 2011 (blue edition, Oberkochen, Germany) software.

For time-lapse microscopy, 501mel cells were grown in glass bottom dishes (Greiner Bio-One, CeLLview, 627870, Frickenhausen, Germany) and transiently transfected with OTC-GFP as outlined above (Table 1). The time series consisted of 50 cycles with 2 second intervals. A cellular region was bleached with the 561 nm excitation wavelength (attenuation set to 10%), which was simultaneously used to activate hypericin. Bleaching of the region of interest was set after 25 of 50 scans, with 50 iterations. Only the 561 nm excitation was used, to minimize hypericin activation from other wavelengths. Controls included vehicle-treated cells.

### Super-resolution structured illumination microscopy (SR-SIM)

The 501mel melanoma cells were seeded onto 35 mm^2^ microscope cover glasses, grown to 80% confluency and transiently transfected with pEYFP-ER (calreticulin) and pYFP-LAMP1, using TransFectin (1 µl, Biorad, 170-3351, Hercules, CA, USA) according to the manufacturer's instructions (Table 1). Controls of the parental plasmids were included. At 20 hours after transfection, the cells were exposed to 3 µM hypericin for 4 hours, followed by light-activation. Complete media was added to the cells after hypericin-PDT and cells were fixed at 30 and 60 minutes post PDT. A vehicle-treated, sham-irradiated control (Hypericin −Light) was included, which was fixed immediately after light-activation. Cells were fixed with 4% paraformaldehyde for 20 minutes, washed in 1× PBS and mounted onto glass slides in moviol mounting medium containing N-propylgalate.

Slides were prepared as described above and super-resolution structured illumination (SR-SIM) was performed. Thin (0.1 µm) z-stacks of high-resolution image frames were collected in 5 rotations by utilizing an alpha Plan-Apochromat 100×/1.46 oil DIC M27 ELYRA objective, using an ELYRA S.1 (Carl Zeiss Microimaging microscope equipped with a 488 nm laser (100 mW), 561 nm laser (100 mW) and Andor EM-CCD camera (iXon DU 885, Oberkochen, Germany). Images were reconstructed using ZEN software (black edition, 2011, version 7.04.287, Oberkochen, Germany) based on a structured illumination algorithm [50]. Analysis was performed on reconstructed super-resolution images in ZEN. Experiments were conducted twice (n = 2).

### Flow cytometry

Flow cytometry was employed to analyze phosphatidylserine (PS) exposure using Annexin V-FITC staining and loss of cell membrane integrity using violet amine reactive viability dye (VIVID) staining [51], [52]. Briefly, cells were seeded at a density of 5×10^4^ in 35 mm tissue culture dishes and allowed to adhere overnight. Cells were then treated with hypericin-PDT and analyzed at 30 min, 1, 4, 7 and 24 hours after treatment. For the 24 hour time-point, cells were seeded and treated a day earlier. Controls included unstained cells and untreated cells (i.e. not sham-irradiated or vehicle-treated). All processing was done on ice. At the respective time-points the culture medium was collected, pooled with the suspended cells and centrifuged for 5 min at 1200 rpm to ensure that all the cellular material was collected. Resulting cell pellets were re-suspended in ice-cold 1× PBS and centrifuged for 5 min at 1200 rpm. The pellet was re-suspended in 500 µl ice-cold 1× PBS, to which 0.5 µl VIVID (Invitrogen Molecular Probes, L34955, Carlsbad, CA, USA) was added. Cells were stained on ice for 30 min, centrifuged for 5 min at 1200 rpm, re-suspended in 1 ml of 1% BSA in 1× PBS and re-centrifuged for 5 min at 1200 rpm. Cells were then re-suspended in 200 µl 1× Annexin V binding buffer to which 1 µl Annexin V-FITC (BD Biosciences, 556420, Franklin Lakes, New Jersey, USA) was added. After 15 min at room temperature, samples were transferred to 5 ml FACS tubes (BD Biosciences, REF352052, Franklin Lakes, New Jersey, USA) and 10,000 events were acquired on a LSRII flow cytometer (BD Biosciences, Franklin Lakes, New Jersey, USA).

The following LSRII parameters were used: blue 488 nm laser 530/30 505LP (detection of FITC); violet 405 nm laser 440/40 (detection of VIVID); voltages: FITC 520, VIVID 370, forward scatter (FS) 250 and side scatter (SS) 320. Unstained cells and single-stained mouse κ beads (BD Biosciences, 552844, Franklin Lakes, New Jersey, USA) were used as controls and to calculate compensation for every run. The beads were stained in 200 µl PBS with 5 µl of the following antibodies: Anti-Human IL-2-FITC (BD Biosciences, 340448, Franklin Lakes, New Jersey, USA) for Annexin V and mouse anti-human CD3-Pacific Blue (BD Biosciences, 558117, Franklin Lakes, New Jersey, USA) for VIVID. Cells were gated on the vehicle-treated, sham-irradiated control (Control −light). Data was analyzed with FlowJo version 7.6.5 (TreeStar, Ashland, USA).

### Western blotting

For preparation of whole cell lysates, cells were grown in 6 cm tissue culture dishes to 90% confluency and treated with 3 µM hypericin-PDT. At 1, 4, 7 and 24 hours after treatment, suspended cells were collected and pooled with adherent cells harvested by scraping in 70 µl complete extraction buffer (100 µM Tris HCL, 1% Nonidet P-40, 0.01% SDS, 0.001 mg/ml Aprotinin and 0.1 mM PMSF, Sigma, St. Louis, MO, USA) using a rubber syringe stopper. Resulting cell lysates were added to 1.5 ml Eppendorf tubes, vortexed briefly and incubated overnight at 4°C. The following day, the cell lysates were centrifuged at 4°C for 20 min at 12000 rpm, the supernatants frozen in aliquots at −80°C and protein concentrations determined using the BCA assay according to manufacturer's instructions (Thermo Scientific Pierce, BCA protein assay kit, 23225, Waltham, Massachusetts, USA).

To separate the various proteins in the cell extracts, equal amounts of proteins (25 µg) were loaded onto a resolving SDS-polyacrylamide gel and electrophoresed at 80 V for 30 minutes followed by 120 V for approximately 2.5 hours in running buffer (0.025 M Tris, 0.192 M Glycine, 0.01% SDS). The proteins were then transferred onto a nitrocellulose membrane (Hybond ECL Amersham, RPN203D, GE Healthcare, Fairfield, CT, USA) in transfer buffer (0.031 M Tris, 0.192 M Glycine, 20% Methanol) at 50 V for 2 hours. The blotted membrane was then immersed in sufficient PonceauS (0.1% Ponceau S, 1% acetic acid) and stained for total protein for 5 minutes. After staining the membrane was immersed in an aqueous solution containing 5% acetic acid for 5 minutes, the solution was then changed and the membrane immersed for another 5 minutes. The membrane was then washed in water (2×5 min). The membrane was scanned digitally and washed twice in TBS-Tween (TBS-T, 0.05 M Tris, 0.15 M NaCl, 0.1% Tween, pH 7.5) for 5 minutes. It was then blocked in 5% milk TBS-T for 1 hour at room temperature. After blocking, the membrane was exposed to primary antibody (AIF 1∶8000 (Sigma, A7549, St. Louis, MO, USA), CASP3 1∶1000 (Cell Signaling Technology, 9661, Danvers, Massachusetts, USA), CASP8 1∶1000 (Cell Signaling Technology, 9746, Danvers, Massachusetts, USA), PARP1 1∶2000 (Santa Cruz Biotechnology, sc-7150, Dallas, Texas, USA) overnight at 4°C. The following day the membrane was washed in TBS-T (3×5 min) and the appropriate secondary antibody was added (GAM-HRP 1∶3000 (AIF), 1∶1500 (CASP8, Bio-Rad, 170-6516, Hercules, CA, USA), GAR-HRP 1∶1500 (CASP3, PARP1, Bio-Rad, 170-6515, Hercules, CA, USA) for 1 hour at room temperature. Visualization of the signal was obtained using SuperSignal West Pico chemiluminescent ECL detection reagent (Thermo Scientific Pierce, Waltham, Massachusetts, USA) with resultant photographic development (AGFA,G150, Mortsel, Belgium) and fixation (AGFA, G354, Mortsel, Belgium). Resulting films were scanned and analysed densitometrically. The total protein Ponceau S stained membrane was used as the loading control [53], [54]. The optical densities (OD) of the exposed film bands of the protein of interest were quantified using ImageJ (National Institutes of Health, USA) and normalized to the OD of the total protein loading control. The resulting ratio was furthermore normalized to the vehicle-treated, sham-irradiated control (Control −light) of the respective experiment. Cells treated with 5 µM doxorubicin for 24 hours were included as a positive control and subjected to identical protocols.

### Data analyses

All data was presented and analyzed using Graphpad Prism (Version 5, Graphpad Software Inc., La Jolla, CA, USA). Statistical analyses were performed by One-Way Anova with Bonferroni post-test (comparing all groups) or Dunnett post-test (comparing all groups to a control group). Differences in values were stated as significant if the p-value was less than 0.05 (p<0.05). All experiments were conducted at least 3 times and presented as mean±SEM, unless otherwise indicated.

### Ethics Statement

The 501mel and the UCT Mel-1 melanoma cells were gifts from Prof. Sharon Prince, Dept of Human Biology, University of Cape Town and Dr EL Wilson, Dept of Haematology, Groote Schuur Hospital, Cape Town, respectively. Both these cell lines were obtained through written, informed consent approved by the Institutional Research Ethics Committee (University of Cape Town).

## Results

Both pigmented (UCT Mel-1) and unpigmented (A375, 501mel) melanoma cells revealed a dose-dependent susceptibility to hypericin-PDT in our previous *in vitro* studies [21], [22] with a dose of 3 µM light-activated hypericin significantly reducing melanoma cell viability to 50% or less than the control. We thus settled on this dose as an effective killing-dose in all subsequent investigations, including those reported in this study. Although this sub-lethal killing-dose is below the desired 90–99% effective clinical killing-dose, it was necessary to employ this dose to be able to study the effects of hypericin-PDT on the surviving cells.

### Phenotypic heterogeneity evident in melanoma cells

**Figure 1:**
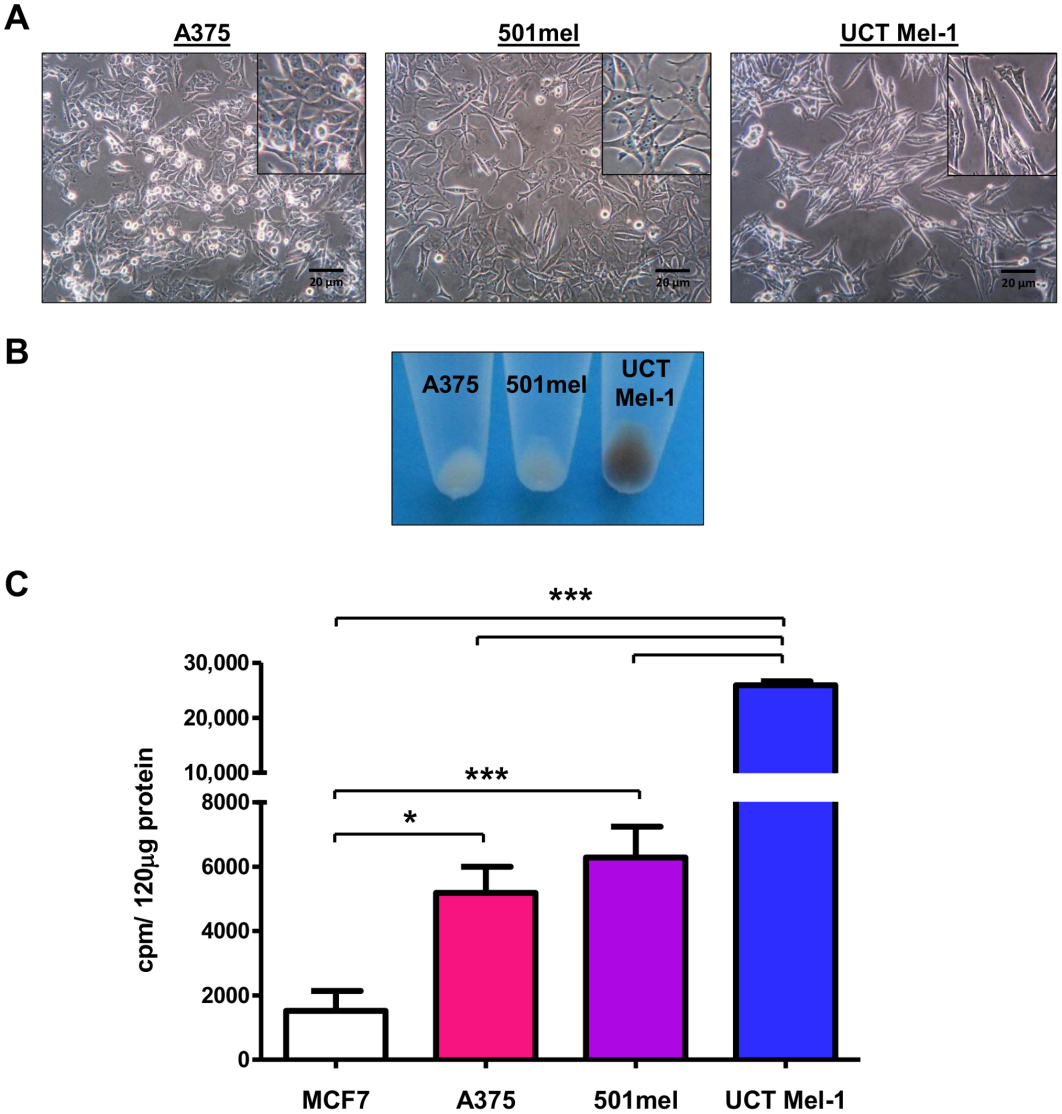
Phenotypic heterogeneity of melanoma cells. (A) Phase contrast images. A representative result is shown (n≥3, scale bars: 20 µm, inset: higher magnification). (B) Cell pellets. A representative result is shown (n≥3). (C) Mean±SEM tyrosinase activity in counts per minute (cpm)/120 µg protein (n = 3, ***p<0.0001, *p<0.05). MCF7 breast cancer cells were included as a negative control.

The three metastatic melanoma cell lines used in this study displayed heterogenous phenotypes. The A375, 501mel and UCT Mel-1 cells presented with epitheloid, multipolar stellate and spindle-shaped morphologies, respectively (Fig. 1A). These cells also exhibited similar growth rates to each other in vitro with a higher average growth rate than their normal counterparts, primary human melanocytes (data not shown). Correlating to the difference in pigmentation observed from the color of the pellets (Fig. 1B), quantification of intracellular tyrosinase activity showed significant differences between the highly pigmented UCT Mel-1 cells and both the A375 (∼5-fold less) and 501mel (∼4-fold less) cells (Fig. 1C). Significant differences also existed between all the melanoma cells and the non-melanoma breast cancer cell line, MCF7, used as a negative control (Fig. 1C).

### Hypericin was taken up by melanoma cells and localized to several intracellular organelles

**Figure 2:**
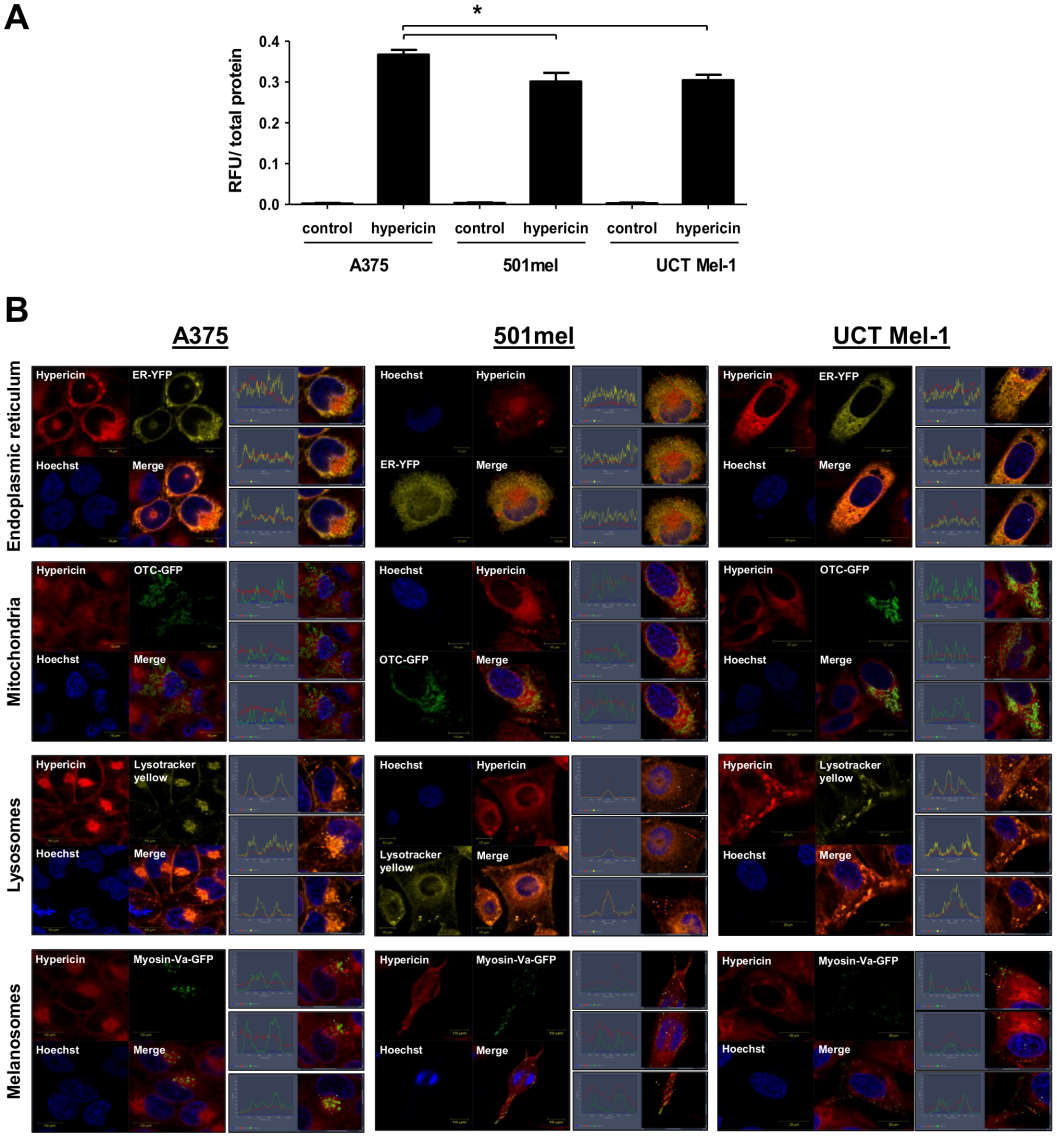
Hypericin uptake and intracellular localization. Cells were exposed to 3 µM hypericin for 4 h without light activation. (A) Hypericin uptake assay. Data is shown as mean±SEM relative fluorescent units per microgram of protein (RFU/µg of protein, n = 3, *p<0.05). (B) Live confocal fluorescent microscopy images of melanoma cells indicate the intracellular localization of hypericin (red) in relation to the endoplasmic reticulum (ER-YFP), mitochondria (OTC-GFP), lysosomes (Lysotracker yellow) and mature melanosomes (MyosinVa-GFP). Nuclei were counterstained with Hoechst (blue). Profiles taken at different locations through the cell indicate co-localization of the fluorophores. A representative result is shown (n = 3, scale bars: 10/20 µm).

The photosenzitizer, light and oxygen constitute the ‘trinity’ of photodynamic therapy (PDT). Effective cellular uptake and intracellular localisation of the photosensitizer is thus crucial for PDT. Hypericin is a naphtodianthrone with a wide absorbance spectrum and characteristic red fluorescence (Ex: 548/593, Em: 594/642) [55]. This fluorescent characteristic was used to relatively quantify hypericin inside the cell using relative fluorescent units (RFU) per microgram of total cellular protein as read on a fluorimeter [48]. All treated cells (3 µM hypericin for 4 hours) displayed intracellular hypericin fluorescence, with 0.37±0.01 RFU/total protein for the A375 cells, significantly higher than 501mel (0.30±0.01 RFU/total protein) and UCT Mel-1 (0.30±0.01 RFU/total protein, Fig. 2A). Hypericin fluorescence in 501mel and UCT Mel-1 was not significantly different from each other (Fig. 2A). Control cells (not treated with hypericin) showed no fluorescence (Fig. 2A). Hypericin was therefore effectively taken up by melanoma cells after 4 hours of exposure.

The principle of PDT is the primary production of reactive oxygen species (ROS) which have short half-lives and small radii of diffusion, subsequently eliciting their action in the vicinity of their production [25], [26]. This calls for the investigation of the intracellular localisation of a photosensitizer as it inevitably affects its mechanism of action [27]. We transiently expressed organelle-specific G and/or YFP-plasmids and stained the cells with probes to fluorescently label different intracellular organelles in order to visualize hypericin localisation. Cells were exposed to 3 µM hypericin for 4 hours without light activation and fluorophores were visualized with live confocal fluorescent microscopy. Controls including G/YFP parental plasmid transfection or probes by themselves were included to test the experimental system employed and revealed no cytotoxicity or aberrant targeting. Hypericin partially co-localized with the endoplasmic reticulum and mitochondria of melanoma cells (Fig. 2B, Fig. S1). Although confocal microscopy profiles taken through the cells at different locations did not show exact matching of these fluorophores, a large overlap between the G/YFP fusion proteins and hypericin signal was found, as visualized by the overlap of the two channels (Fig. 2B, Fig. S1). To visualize lysosomes, melanoma cells were exposed to Lysotracker yellow (Fig. 2B). This fluorescent acidotropic probe labels acidic organelles in live cells. This includes lysosomes, late stage endosomes and possibly melanosomes, the pigment producing, acidic organelles. Confocal profiles revealed co-localization of hypericin with lysosomes/late stage endosomes and melanosomes in all melanoma cells (Fig. 2B). To further investigate melanosomal co-localization, mature melanosomes were visualized in melanoma cells using specific GFP fusion proteins. Partial hypericin co-localization with mature melanosomes was found in 501mel and UCT Mel-1 cells, whereas no co-localization was evident in A375 cells (Fig. 2B). These results were further confirmed using other early and mature melanosomal markers (Fig. S1). Noteworthy, hypericin did not co-localize with nuclei in any of the melanoma cells investigated.

### Hypericin-PDT induced photodestruction of organellar structure

**Figure 3:**
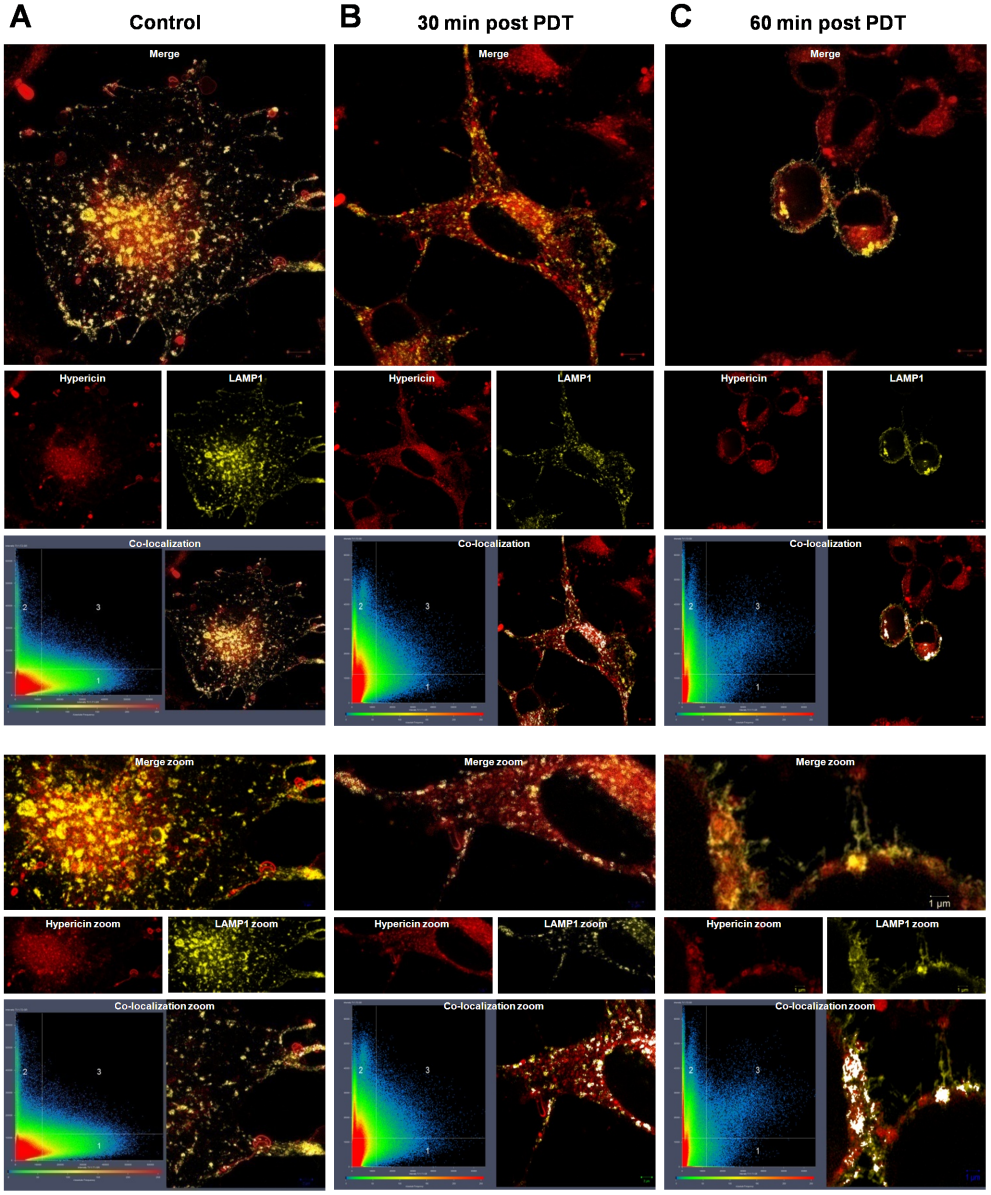
Hypericin-PDT induced loss of structural details of LAMP1 positive structures (endosomes, lysosomes and melanosomes). Cells expressing LAMP1-YFP were exposed to 3 µM hypericin (red) for 4 h, followed by light-activation and imaging using Super-resolution structured illumination microscopy (SR-SIM). (A) Control (hypericin-treated, sham-irradiated). (B) 30 min post PDT. (C) 60 min post PDT. Images are shown at lower magnification (top panel, scale bars: 5 µm) and higher magnification (zoom, lower panel, scale bars: 1/2 µm.) Co-localization plots indicate co-localization of the fluorophores. A representative result is shown (n = 2).

**Figure 4:**
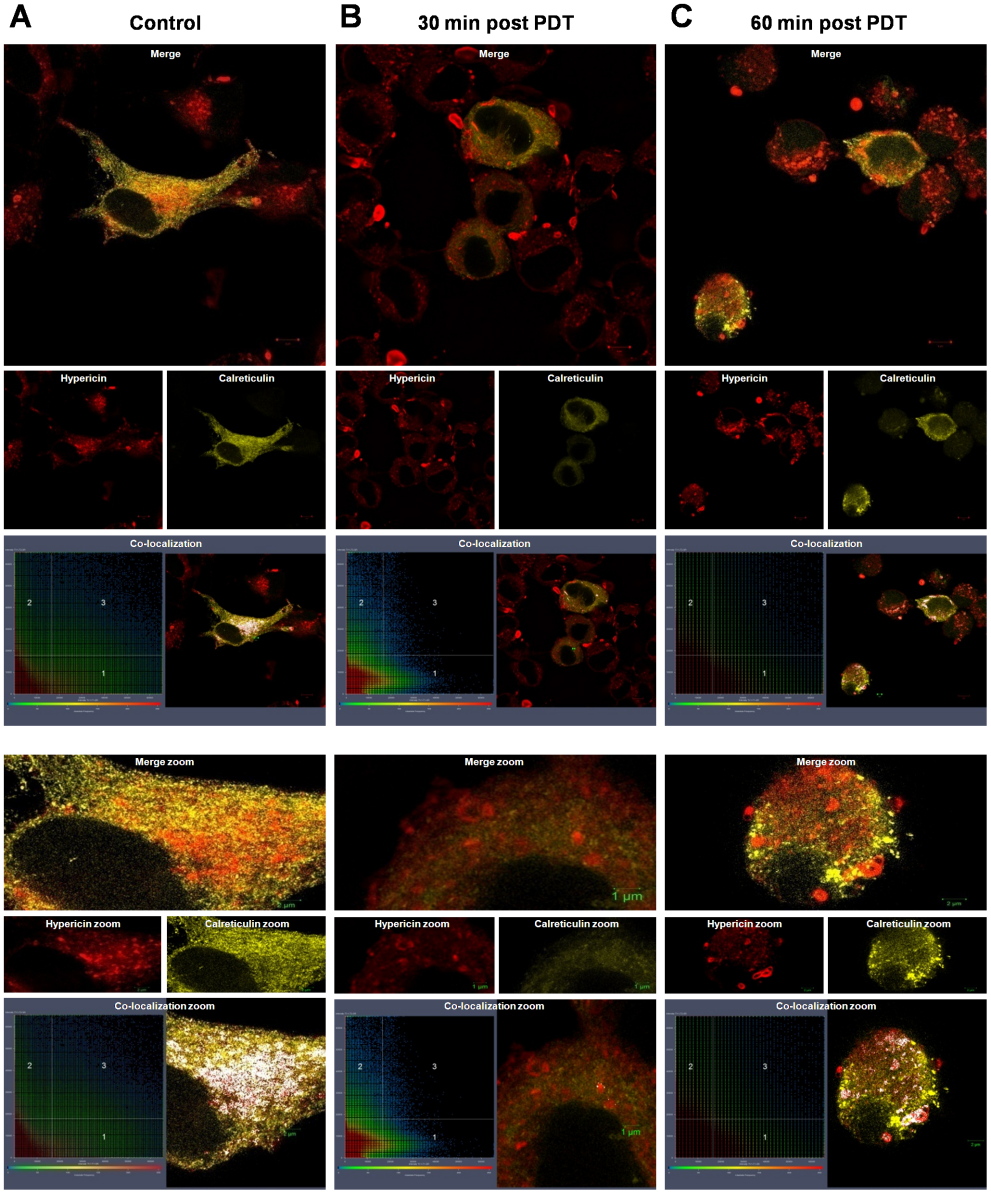
Hypericin-PDT induced loss of structural details of calreticulin positive structures (endoplasmic reticulum). Cells expressing calreticulin-YFP (ER-YFP) were exposed to 3 µM hypericin (red) for 4 h, followed by light-activation and imaging using Super-resolution structured illumination microscopy (SR-SIM). (A) Control (hypericin-treated, sham-irradiated). (B) 30 min post PDT. (C) 60 min post PDT. Images are shown at lower magnification (top panel, scale bars: 5 µm) and higher magnification (zoom, lower panel, scale bars: 1/2 µm.) Co-localization plots indicate co-localization of the fluorophores. A representative result is shown (n = 2).

Upon visualization of hypericin in GFP-labeled mitochondria with live confocal fluorescent microscopy, we noticed distinct changes in the mitochondrial structure of melanoma cells. To investigate this in more detail we used the unpigmented 501mel melanoma cells and activated hypericin in real time on the confocal fluorescent microscope using time-lapse technology. The 501mel cells displayed a rapid and extensive modification from its normal tubular mitochondrial network into a beaded appearance upon light-activation of hypericin (Vid. S1). This was not observed in the untreated control cells expressing the mitochondrial-specific GFP fusion protein (Vid. S2). Despite hypericin partially co-localizing with the endoplasmic reticulum and lysosomes in the melanoma cells (Fig. 2A, Fig. S1), rapid photo-destruction was only observed in mitochondria. Whether this is related to an organelle-specific, concentration-dependent effect or simply an energetic, metabolic effect remains to be determined. However, to further investigate the light-activation effect on the structural details of intracellular organelles, we transiently expressed calreticulin and lysosomal-associated membrane protein (LAMP1) in 501mel cells to visualize the endoplasmic reticulum and lysosomes respectively, before and after PDT using super-resolution structured illumination microscopy (SR-SIM). A partial co-localization of hypericin with LAMP1 positive structures (lysosomes, endosomes and melanosomes, Fig. 3A) and calreticulin-positive structures (endoplasmic reticulum, Fig. 4A) was observed before light-activation, confirming the confocal fluorescent microscopy data (Fig. 2, Fig. S1). Surprisingly, an increased co-localization of hypericin with LAMP1 positive structures was found at 30 and 60 minutes post-PDT, whilst the structure of these organelles remained intact (Fig. 3B, C). In contrast, a loss in structural detail for calreticulin-positive structures was found post-PDT, suggestive of organelle disruption, which was associated with a loss in co-localization of hypericin and calreticulin-positive structures (Fig. 4B, C).

### Hypericin-PDT induced phosphatidylserine exposure and loss of melanoma cell membrane integrity

A change in cell membrane structure by surface exposure of phosphatidylserine (PS) is observed in early apoptotic cells [52], [56]. Using Annexin V in conjunction with cell permeability dye (e.g. propidium iodide), enables the distinction between cells with exposed PS and those with compromised cell membranes. However, due to the red, fluorescent nature of propidium iodide, it was not the most suitable assay to use in conjunction with the red, autofluorescent hypericin. To prevent confounding results, we thus used the violet amine reactive viability dye (VIVID), to investigate loss of cell membrane integrity in our experimental system [51].

**Figure 5:**
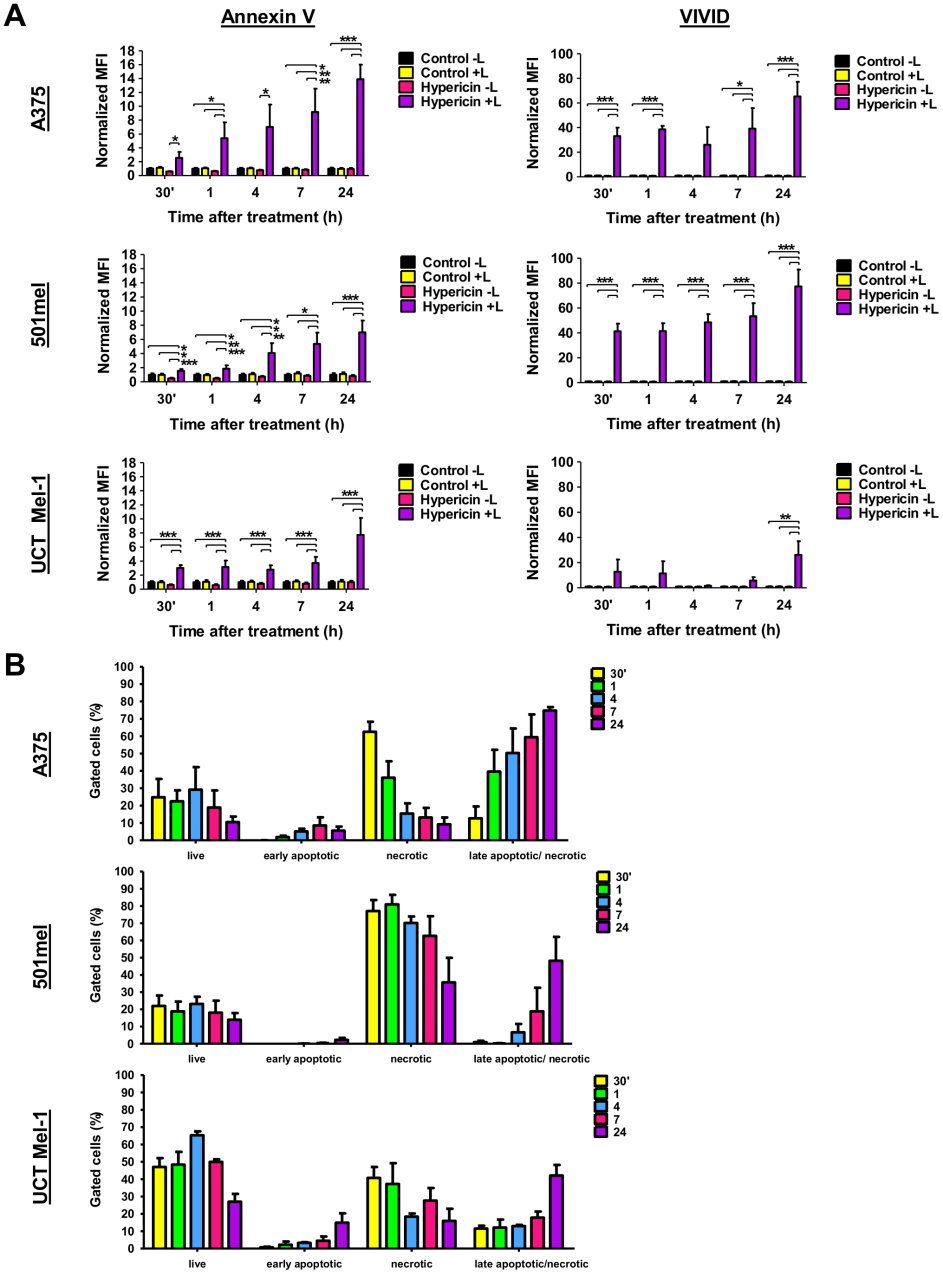
Hypericin-PDT induced phosphatidylserine exposure and loss of cell membrane integrity. (A) Annexin V (phosphatidyl serine exposure) and VIVID (loss of cell membrane integrity) median fluorescent intensities (MFI) normalized to the vehicle-treated, sham-irradiated control (Control −Light) at 30 min, 1, 4, 7 and 24 h after treatment. Flow fluorocytometric data is shown as the median±SEM (n≥3, ***p<0.0001, **p<0.01, *p<0.05, L: light). (B) Percentage gated cells of 4 different populations labeled with Annexin V and VIVD: live (AV− VIVD−), early apoptotic (AV+ VIVID−), necrotic (AV− VIVID+) and late apoptotic/necrotic (AV+ VIVID+) at 30 min, 1, 4, 7 and 24 h after treatment. Data is shown as mean±SEM of gated cells (n≥3).

Melanoma cells were treated with 3 µM hypericin and analysed for PS exposure (Annexin V) and loss of cell membrane integrity (VIVID), 30 minutes, 1, 4, 7 and 24 hours after treatment using fluorescent activated cell sorting (FACS). Cells treated with vehicle with sham-irradiation (Control –light) displayed intact cell membranes with no evidence of PS externalization (Fig. 5A, left panel). The Annexin V median fluorescent intensity (MFI) analyses revealed a time-dependent increase in PS exposure after light-activated hypericin treatment in all melanoma cells, significantly different to controls (Fig. 5A, left panel). The highest Annexin V MFI was observed at 24 hours after treatment, most pronounced in A375 (14.1±1.5 fold) and similar in 501mel (7.2±1.0 fold) and UCT Mel-1 (7.5±1.3 fold), compared to that of the vehicle-treated, sham-irradiated control (Control –light, Fig. 5A, left panel).

The VIVID MFI analyses showed loss of 501mel cell membrane integrity after light-activated hypericin treatment at all time-points investigated, significantly different to the controls (Fig. 5A, right panel). The A375 cells also lost membrane integrity at all time-points except for the 4 hour time-point which showed a maintenance of cell membrane integrity (Fig. 5A, right panel). In contrast, UCT Mel-1 cell membrane integrity stayed intact up to 7 hours (Fig. 5A, right panel). These changes in cell membrane permeability were most pronounced at 24 hours for all cells (A375: 81.5±9.4, 501mel: 69.2±11 and UCT Mel-1: 25.3±6.1 fold compared to control, Fig. 5A, right panel).

Flow fluorocytometric analyses of Annexin V versus VIVID further enabled the identification of 4 different populations: live (Annexin V− VIVID−), early apoptotic (Annexin V+ VIVID−), early necrotic (Annexin V− VIVID+) and late apoptotic/necrotic (Annexin V+ VIVID+) (Fig. 5B).

The live population for UCT Mel-1 cells was approximately 2-fold higher than the A375 and 501mel cells at all time-points investigated (Fig. 5B). The A375 and 501mel live populations were similar to each other. These results clearly suggest that UCT Mel-1 cells are more resistant to hypericin-PDT than A375 or 501mel cells. Control treatments all resulted in a live population of approximately 95% or more for all melanoma cells (Fig. S2).

Death population profiling (Fig. 5B) showed that the A375 melanoma cells displayed an initial early necrotic response at 30 minutes after treatment (63±6%), followed by a time-dependent decrease in the early necrotic population to 9±4% at 24 hours (Fig. 5B). Conversely, the late apoptotic/necrotic population increased with time after treatment from an initial 13±7% at 30 minutes to 75±12% at 24 hours (Fig. 5B).

Treatment of 501mel cells with light-activated hypericin resulted in a predominantly early necrotic population (Fig. 5B). This population was highest at 30 minutes after treatment (77±6%) with comparable values up to 7 hours. At 24 hours, 501mel cells showed similar early necrotic (36±14%) and late apoptotic/necrotic populations (48±14%, Fig. 5B). At time points earlier than 24 hours the late apoptotic/necrotic population was low (4 h: 7±5%, 7 h: 19±14%, Fig. 5B).

UCT Mel-1 cells displayed an initial early necrotic response (41±6%) at 30 minutes after light-activated hypericin treatment (Fig. 5B) which decreased with time (24 h: 16±7%). The late apoptotic/necrotic population was initially low (30 min: 12±2%) and only increased by 24 hours (42±6%). At 24 hours UCT Mel-1 cells showed an early apoptotic population of 15±5% (Fig. 5B).

One common feature of the cell death response to hypericin-PDT for all melanoma cell lines investigated was minimal early apoptotic induction. UCT Mel-1 displayed the highest early apoptotic population with 15±5% at 24 hours, followed by 9±5% for A375 at 7 hours and 2±1% for 501mel at 24 hours (Fig. 5B).

In summary, it was clear from this data that melanoma cells died by specific mechanisms involving the externalization of PS and loss of cell membrane integrity at specific times after hypericin-PDT treatment and this death induction was melanoma cell type and time-dependent.

### Hypericin-PDT reduced cellular size and increased cellular granularity/pigmentation

Morphological alterations of dying cells include shrinkage of apoptotic cells and swelling of necrotic cells [57]. Both these morphological alterations can be quantified as forward scatter (FS, cell size) and side-scatter (SS, granularity) and has been used in various experimental systems to identify cell populations with different pigmentation patterns [58]–[60]. In addition, melanoma cells have been shown to use their pigmentary system to sequester chemotherapeutics into melanosomes and through melanogenesis circumvent cell death [61]–[63].Furthermore, melanin and melanogenesis, besides serving as markers of melanoma differentiation, also indicate the production of resistance to therapy [3], [64]. Moreover, melanin has also been shown to act as a radioprotector [65]. It is thus pertinent to identify whether pigmentation plays a protective or destructive role in hypericin-PDT.

**Figure 6:**
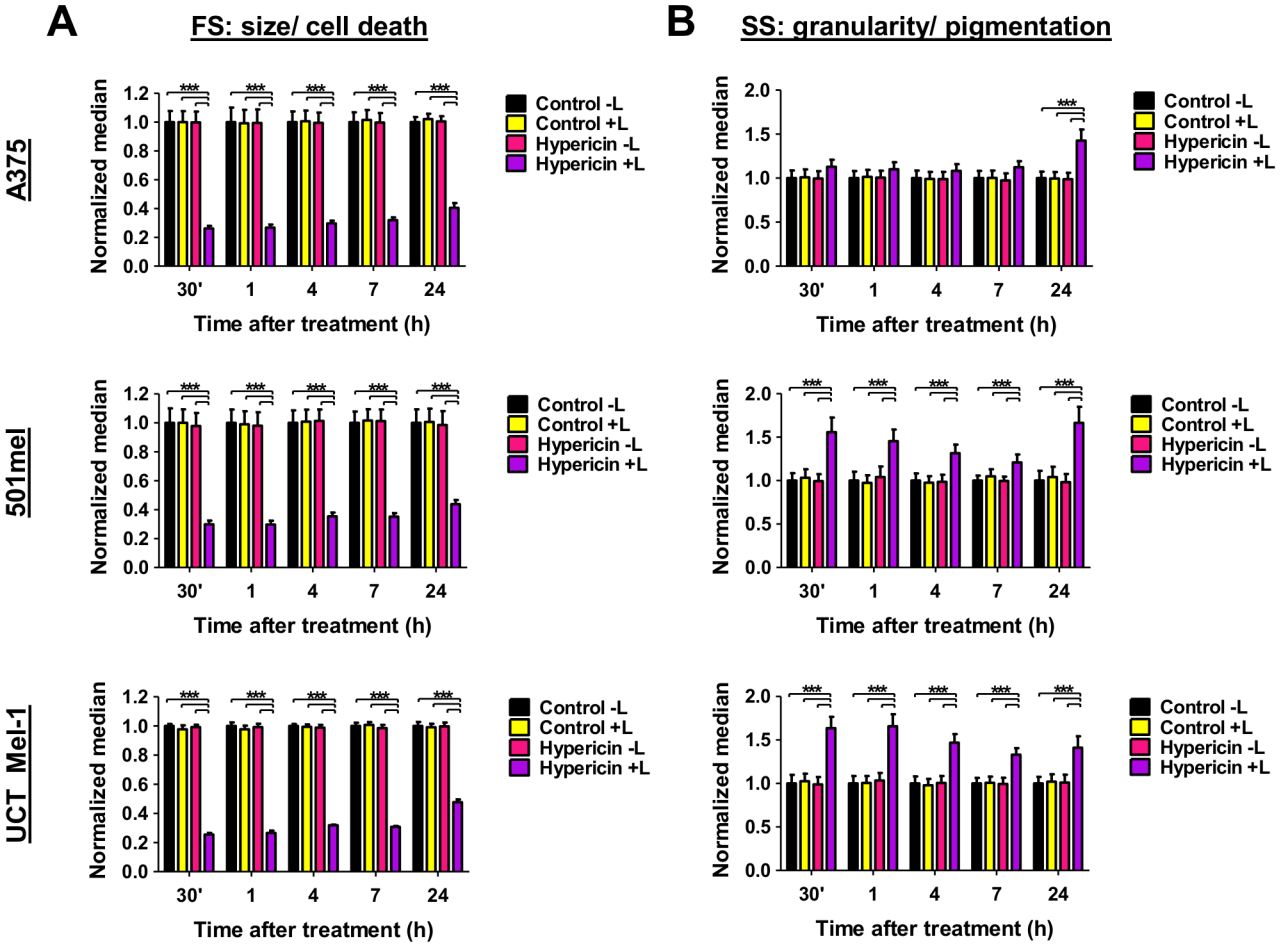
Hypericin-PDT reduced cellular size and increased cellular granularity/pigmentation. (A) Melanoma cell forward scatter (FS) as an indication of cell size/cell death mechanisms and (B) melanoma cell side scatter (SS) as an indication of cell granularity/pigmentation; normalized to the vehicle-treated, sham-irradiated control (Control −Light) at 30 min, 1, 4, 7 and 24 h after treatment. Flow cytometric data is shown as median±SEM (n≥3, ***p<0.0001, L: light).

Light-activated hypericin treatment reduced melanoma cell size (FS) to approximately a third of that of the control cells, for all melanoma cell lines investigated at all time-points after treatment (Fig. 6A). At 24 hours after treatment cells were slightly larger compared to the other time points but still significantly smaller than controls (A375: 0.40±0.02, 501mel: 0.44±0.03 and UCT Mel-1: 0.48±0.01 fold, Fig. 6A).

A significant increase in granularity/pigmentation (SS) compared to the control was observed at all time-points after light-activated hypericin treatment of 501mel and UCT Mel-1 cells (Fig. 6B). This was most pronounced at 24 hours for both 501mel (1.67±0.05 fold) and A375 cells but at 1 hour for UCT Mel-1 (1.66±0.04 fold). The A375 granularity/pigmentation significantly increased at 24 hours to 1.42±0.03 fold of the control, with no significant change at the other time-points investigated (Fig. 6B).

### Hypericin-PDT induced the expression of apoptotic proteins

To shed more light on hypericin-PDT induced cell death mechanisms we further investigated the involvement of specific cell death proteins. These included: caspase 3 (CASP3), caspase 8 (CASP8), apoptosis inducing factor (AIF) and poly(ADP-ribose)polymerase 1 (PARP1).

A vital step in both the intrinsic and extrinsic caspase-dependent apoptotic cascade is the activation of the executioner CASP3 [66]. The initiator caspase of the extrinsic apoptotic pathway is CASP8, which upon activation either activates CASP3 directly or indirectly through activation of the mitochondrial apoptotic pathway [67]. The intracellular apoptotic pathway can however also be executed in a caspase-independent manner, through factors such as AIF [68]. Poly(ADP-ribose)polymerase 1 (PARP1) is involved in both parthanatos with AIF [69] and apoptosis with caspases, as it contains a caspase cleavage site [70].

**Figure 7:**
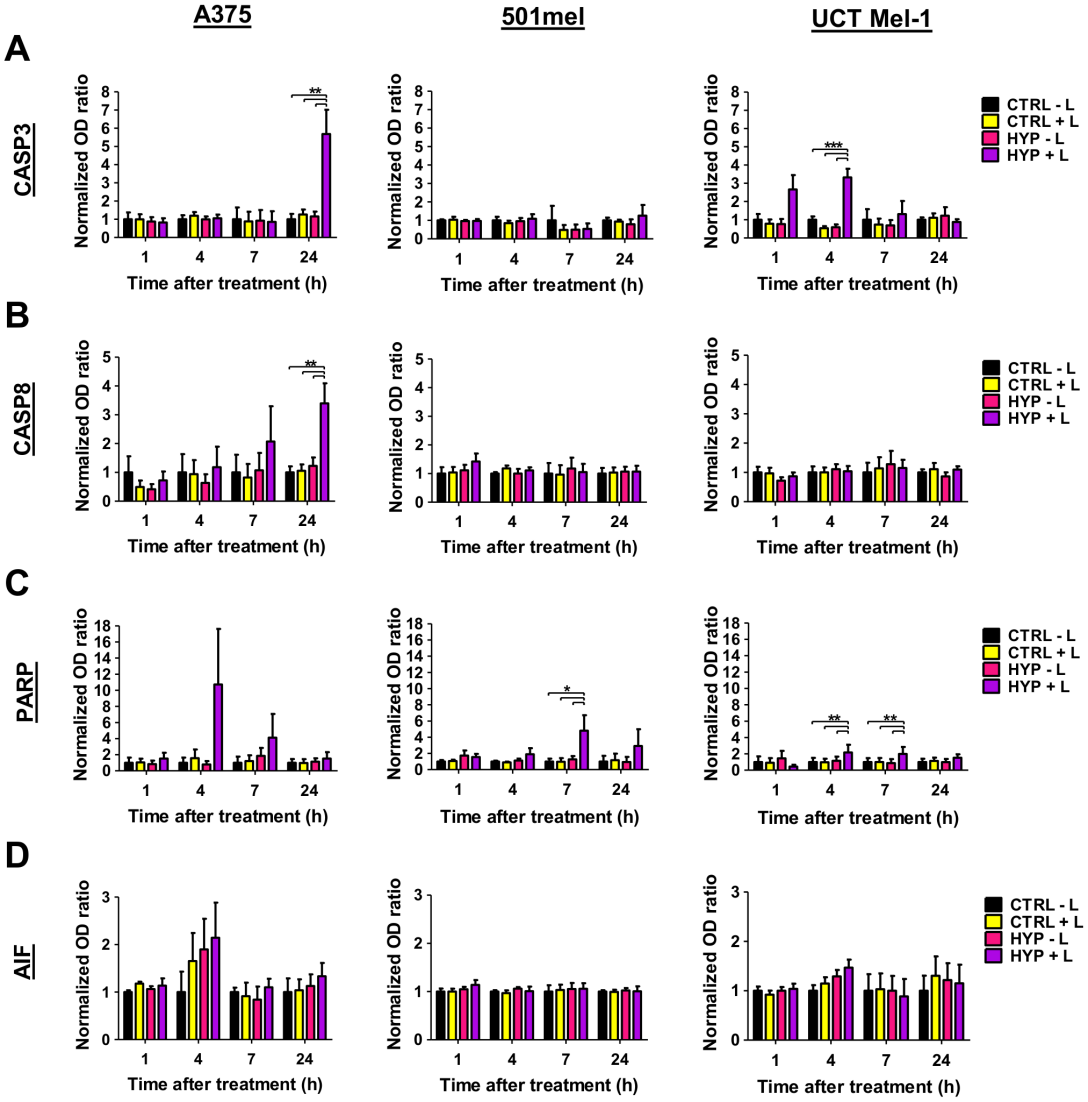
Hypericin-PDT induced expression of apoptotic proteins. (A) Caspase 3 (CASP3), (B) caspase 8 (CASP8), (C) poly(ADP-ribose)polymerase 1 (PARP1) and (D) apoptosis inducing factor (AIF) Western blot analyses of whole cell lysates detected at 1, 4, 7 and 24 h after treatment. Data is shown as mean±SEM normalized OD ratio (n≥3, ***p<0.0001, **p<0.01, *p<0.05, CTRL: vehicle-treated control, HYP: hypericin and L: light).

Melanoma cells were treated with 3 µM light-activated hypericin followed by Western blot analyses at 1, 4, 7 and 24 hours after treatment. Resulting data was normalized to total protein used as a loading control and ratios to the vehicle-treated and sham-irradiated control (Control −light) were densitometrically quantified and presented (Fig. 7, representative results of x-ray films can be found in Fig. S3).

Hypericin-PDT elicited a differential cell death response in melanoma cells (Fig. 7). The A375 melanoma cells initiated extrinsic apoptosis at 24 hours post hypericin-PDT, evident by the activation of the initiator CASP8 (Fig. 7B) and executioner CASP3 (Fig. 7A). The intrinsic apoptotic cascade was activated in UCT Mel-1 melanoma cells at 4 hours after treatment with the activation of CASP3 (Fig. 7A) and cleavage of PARP, inactivation of PARP was also found at 7 hours (Fig. 7C). The 501mel cells expressed cleaved PARP at 7 hours post PDT (Fig. 7C); however neither CASP3 nor CASP8 (Fig. 7A/B) were cleaved at any of the time-points investigated, suggesting a CASP3-independent PARP cleavage mechanism. The 501mel cells presented cleaved CASP3 after doxorubicin treatment (used as a positive control), thus indicating that these cells are able to elicit caspase-dependent apoptotic cell death. The lethal form of AIF was not induced by hypericin-PDT in the melanoma cell lines investigated (Fig. 7D). These findings are summarized in Table 2.

**Table 2:** Cell death protein expression in response to hypericin-PDT.

Cell line	A375				501mel				UCT Mel-1			
Time (h)	1	4	7	24	1	4	7	24	1	4	7	24
**CASP3**	−	−	−	+	−	−	−	−	−	+	−	−
**CASP8**	−	−	−	+	−	−	−	−	−	−	−	−
**AIF**	−	−	−	−	−	−	−	−	−	−	−	−
**PARP**	−	−	−	−	−	−	+	−	−	+	+	−

Melanoma cells were treated with 3 µM light-activated hypericin and analyzed for protein expression of caspase 3 (CASP3), caspase 8 (CASP8), apoptosis inducing factor (AIF) and poly(ADP-ribose)polymerase 1 (PARP1) at 1, 4, 7 and 24 hours after treatment by Western blot analyses (−: uncleaved, +: cleaved).

## Discussion

The ‘trinity’ of PDT comprises a photosensitizer, light and molecular oxygen [12]. The choice of the light source in the clinic depends on various factors such as photosensitizer absorption, disease (size, location, accessibility and tissue characteristics) and cost [12]. The penetration of light into tissue increases with its wavelength, therefore photosensitizers with absorption peaks in the red to deep red spectrum (600–800 nm) offer tumour control in deeper tissues [12]. Hypericin absorbs light of both UVA and visible wavelengths [55]. As one of the factors influencing choice of light source for PDT is cost, sunlight-mediated PDT becomes an interesting avenue to explore for the activation of hypericin. It has been shown in 3 randomized controlled studies that daylight-mediated PDT was an effective treatment for thin actinic keratosis [71]–[73]. Daylight-mediated PDT is nearly pain-free, more convenient for both patients and clinics and is especially suited for patients with large field cancerized areas, which can easily be exposed to daylight [74]. It poses a particularly interesting avenue to explore for hospitals in developing countries, such as South Africa, where space and budgets are limited. Overall, it emphasizes that in both cutaneous and metastatic melanoma, hypericin-PDT presents as a good candidate strategy.

On the basis of the above outlined factors, we chose to use UVA to activate hypericin. A dose of 1 J/cm^2^ UVA was employed based on its effective hypericin activation resulting in phototoxicity in experimental systems, including our own [21], [22], [75]. This dose is below the minimal erythemal dose (MED) baseline of 20 J/cm^2^ UVA observed in a study by Beattie et al., 2005 [76].

Hypericin-PDT induced cytotoxicity elicits tumor cell death by various mechanisms including apoptosis, necrosis and autophagy-related cell death [27]–[29]. Additionally, we and others have shown that hypericin-PDT is a potentially effective therapy to reduce melanoma cell viability, through the induction of specific cell death mechanisms thus contributing to increased therapeutic targeting strategies [14], [15], [17]–[19], [21], [22].

### Hypericin-PDT induced the expression of apoptotic proteins

In this study it is shown that hypericin-PDT is effective in killing both unpigmented (A375 and 501mel) and pigmented (UCT Mel-1) melanoma cells through the induction of apoptosis (Fig. 7/Fig. S3). In addition, this treatment resulted in extrinsic (A375) and intrinsic (UCT Mel-1) caspase-dependent as well as a caspase-independent apoptotic mode of cell death, and an apoptotic mode that did not involve AIF (501mel, Fig. 7/Fig. S3). Moreover, each of these mechanisms seem melanoma cell-type specific, reinforcing the heterogenous nature of malignant melanoma cells.

At 4 hours after treatment, CASP3 cleavage was found in UCT Mel-1, whereas in A375 melanoma cells this event occurred at 24 hours (Fig. 7A/Fig. S3). This was not surprising as the involvement of CASP3-mediated cell death in response to hypericin-PDT has been documented in a number of experimental systems [77]–[87]. The positive control used in our experimental system indicated that the 501mel melanoma cells were able to cleave CASP3, but that this CASP3 activation was not induced by hypericin-PDT (Fig. 7A/Fig. S3). Hypericin-PDT also resulted in CASP8 cleavage in the A375 melanoma cells after 24 hours which was not found at any other time points in these cells, or in any of the other cell lines investigated (Fig. 7B/Fig. S3). This hypericin-PDT mediated CASP8 activation has been reported in both Jurkat T-lymphocytes [87] and nasopharyngeal carcinoma cells [81], but not in a variety of murine and human cancer cell lines [84], [85], [88], [89]. To the best of our knowledge, this is the first study to report on this hypericin-PDT mediated activation of CASP8 in melanoma. Whether this is specific to different experimental systems or different cancers is intriguing.

A study by Buytaert et al. (2006), using Bax^−/−^Bak^−/−^ double knockout (DKO) cells suggested that other toxic mitochondrial intermembrane space proteins, such as AIF and cytochrome c, play a role in orchestrating caspase-independent apoptosis by release and direct translocation of these factors from mitochondria to nuclei [90]. This was corroborated in HT-29 adenocarcinoma cells, resulting in AIF-mediated condensation and fragmentation of nuclei in response to hypericin-PDT treatment [91]. In our study, despite hypericin localizing to the mitochondrion and potentially causing mitochondrial outer membrane permeability (MOMP) upon light-activation, there was no evidence for the induced expression of the cleaved, lethal AIF fragment (57 kDa) in any of the melanoma cells (Fig. 7D, Fig. S3), suggesting that AIF-mediated caspase-independent apoptosis are not induced in our experimental system.

In response to DNA damage, PARP1 is activated to facilitate its repair [69]. During apoptotis, PARP1 is cleaved to ensure adequate ATP levels for apoptotic completion [70]. This cleavage is under the control of caspases and results in an 89 and 24 kDa fragment. The 24 kDa fragment irreversibly binds to DNA strand breaks, thereby inhibiting DNA repair enzymes (including PARP1) with a consequent attenuation of DNA repair [92]. The cleavage of PARP1 can furthermore take place by the action of various other suicidal proteases including calpains, cathepsins, granzymes and matrix metalloproteinases [92]. In contrast, overactivation of PARP1 has been implicated in AIF activation, in a regulated necrotic mode of cell death called parthanatos [69]. PARP cleavage has been observed in response to hypericin-PDT treatment [77], [80], [81], [83], [85], [91]. In our system, hypericin-PDT induced PARP cleavage was observed in the pigmented UCT Mel-1 at 4 hours and both the UCT Mel-1 and unpigmented 501mel at 7 hours after treatment (Fig. 7C/Fig. S3). It is probable in the UCT Mel-1 cells, that this cleavage could be mediated by CASP3 as its expression correlated with its cleaved, active form at 4 hours (Fig. 7A/Fig. S3). However, CASP3 cleavage was not evident in both UCT Mel-1 and 501mel cells at 7 hours which suggests the involvement of other suicidal proteases able to cleave PARP1. As we showed partial localization in the ER and lysosomes, the involvement of calpains, cathepsins and apoptosis-inducing proteases cannot be excluded. Significantly, no PARP cleavage was found in the A375 melanoma cells but both CASP8 and CASP3 were cleaved at 24 hours suggesting that perhaps the PARP cleavage occurs at a later, delayed time-point that fell outside the scope of this study. Alternatively the A375 cells may have other DNA protective mechanisms in place delaying the onset of apoptosis. Work is currently underway to explore these possibilities.

### Hypericin-PDT induced phosphatidylserine exposure and loss of cell membrane integrity

A common feature of apoptosis is the change in cell membrane structure by surface exposure of phosphatidylserines (PS), whereas necrosis exhibits a loss of cell membrane integrity [93]. In this study, hypericin-PDT induced the externalization of PS and loss of melanoma cell membrane integrity at specific times after treatment (Fig. 5A). Minimal early apoptotic populations were found in all cell lines (Fig. 5B). However, initial early necrotic populations were also observed in all melanoma cell lines, decreasing over time, followed by a concomitant increase in the late apoptotic/necrotic populations (Fig. 5B). These early necrotic populations of cells were positive for VIVID and negative for Annexin V which is in contrast to Van den Berghe et al. (2013) and others, who described PS exposure and permeabilization of the plasma membrane coinciding with necrosis and an immediate shift from live to late apoptotic/necrotic populations [94], [95]. The subsequent shift from an early necrotic population to a late apoptotic/necrotic population positive for both markers suggests that Annexin V binding in this double-labeled population might be an artifact due to loss of cell membrane integrity. However this was disputed through recent studies that early necrotic cells exposed PS followed by macrophage clearance [96]. A suggestion that the double-labeled population could represent AIF-mediated necroptosis [97]–[100], is unlikely as no associated AIF activation was observed in the melanoma cells (Fig. 7D, Fig. S3).

### Hypericin-PDT reduced cellular size and increased cellular granularity

Recently, the characteristics of melanoma cell size and granularity as expressed by flow cytometric analyses have become a potential measure of the induction of apoptosis. This is based on the externalization of PS through the process of internalization of the cell membrane as cells shrink during apoptosis, forming intracellular vesicles [56]. This was supported by our results (Figs. 6A/B) in which, besides the externalization of PS (Fig. 5A), the melanoma cells decreased in size to less than half of the control at all times after hypericin-PDT treatment for all cell lines (Fig. 6A). Moreover, the reduction in melanoma cell size correlated to an increase in granularity to approximately half of the control (Fig. 6B). We suggest therefore that changes in cellular granularity could indicate changes in pigmentation levels of melanoma cells - a characteristic that has been used in various pigmented experimental systems to identify cell populations with different pigmentation patterns [58]–[60]. Pigmentation in melanoma is related to a melanin-containing, melanocyte-specific organelle, the melanosome, which has recently been implicated in drug trapping, resistance to chemotherapy [62] and hypericin-PDT [17], [18]. The presence or absence of melanin i.e. a melanotic and amelanotic melanoma phenotype, has shown to offer a difference in the sensitivity of melanomas to ionizing and ultraviolet radiation (UVR) suggesting that inhibiting melanogenesis could play a role in melanoma therapy [64], [101].

### Hypericin-PDT induced photodestruction of organellar structure

As the co-localization of hypericin becomes important as a potential contributor to the mode of cell death employed, we analyzed the intracellular localization of hypericin using fluorescent confocal and super-resolution structured illumination microscopy. Hypericin was taken up by all melanoma cells used in this study and partially co-localized to the endoplasmic reticulum (ER), mitochondria, lysosomes and melanosomes, but not the nucleus (Fig. 2, 3, 4, Fig. S1, Vid. S1 & S2). This was not surprising as hypericin has been reported to co-localize with the ER [90], [102]–[106], mitochondria [103], [107], [108] and lysosomes [90], [102], [108]–[110]; eliciting autophagy-related, apoptotic [90], [111], [112] and necrotic [108] cellular responses.

Both lysosomes and melanosomes have been implicated in resistance to cancer therapy. This is not surprising as both these organelles have been shown to have the same ancestral origin [62], [113], [114]. Interestingly, lysosomally targeted photosensitizers have been shown to effectively circumvent multi-drug resistance by lysosomal photodestruction upon light-activation, resulting in the reversion to the parental drug sensitivity [113]. In this study, hypericin only partially co-localized with melanosomal-specific GFP fusion proteins and Lysotracker-positive structures (Fig. 2, Fig. S1), suggesting that these melanoma cells may not be utilizing their melanosomal/lysosomal system for hypericin trapping and export. To shed more light on these phenomena we investigated hypericin co-localization with endosomes, lysosomes and melanosomes (LAMP1 positive structures), in one of the melanoma cell lines (501mel) before and after PDT, using super-resolution structural illumination microscopy (Fig. 3). These results show, for the first time at high resolution in melanoma cells, that the partial co-localization of hypericin and LAMP1 positive structures observed pre-PDT, increased at 30 and 60 minutes post-PDT (Fig. 3). Furthermore, LAMP1 positive structures stayed intact post-PDT (Fig. 3). In contrast, a loss of structural details suggestive of organelle disruption and a decrease in co-localization of hypericin and the ER (calreticulin positive structures) was found post-PDT in 501mel cells (Fig. 4). Moreover, the 501mel cells displayed a rapid, extensive modification of the tubular mitochondrial network into a beaded appearance as shown through live confocal fluorescent time-lapse microscopy (Vid. S1), suggesting a change in mitochondrial function possibly through the disruption of the mitochondrial outer membrane potential (MOMP). The observed loss of structural details of both the ER and mitochondria in response to hypericin-PDT suggests a detrimental effect of ROS production in the vicinity of these structures, leading to organelle disruption. Surprisingly the opposite was found for lysosomal related organelles (LAMP1 positive structures), suggesting that the melanoma cells may be using these LAMP1 positive intracellular organelles for hypericin-PDT resistance. Noteworthy is that these results were obtained at 30 and 60 minutes post-PDT; it will be interesting to investigate later time-points post-treatment in future studies. In line with this speculation we found an increase in cellular granularity, up to 24 hours post-PDT, suggesting an increase in pigmentation levels in these cells in response to hypericin-PDT (Fig. 6B). Moreover, studies from our laboratory have shown that the pharmacological inhibition of tyrosinase resulted in depigmentation of melanoma cells and a concomitant increased susceptibility to hypericin-PDT [17], [18]. In addition, melanin-producing cells have been shown to induce various PDT resistance mechanisms targeting the ‘trinity’ of PDT, the photosensitizer, light and molecular oxygen. These include photosensitizer adsorption inside the polymer, light screening by melanin and oxygen consumption by tyrosinase and melanin itself [11], [64]. Whether melanoma cells use melanosomes/lysosomes to sequester and export hypericin from the cell to counteract PDT, or any of the above mentioned resistance mechanisms of melanin-producing cells, remains to be investigated. The use of hypericin hydroquinone is an interesting avenue to explore in this context, as it could act as a melanogenesis-inhibiting agent as well as a photosensitizer, with the additional advantage of absorbance in the red spectrum offering deeper light activation in the tumour [115]. Further research is needed to shed more light on these mechanisms.

## Conclusions

**Figure 8:**
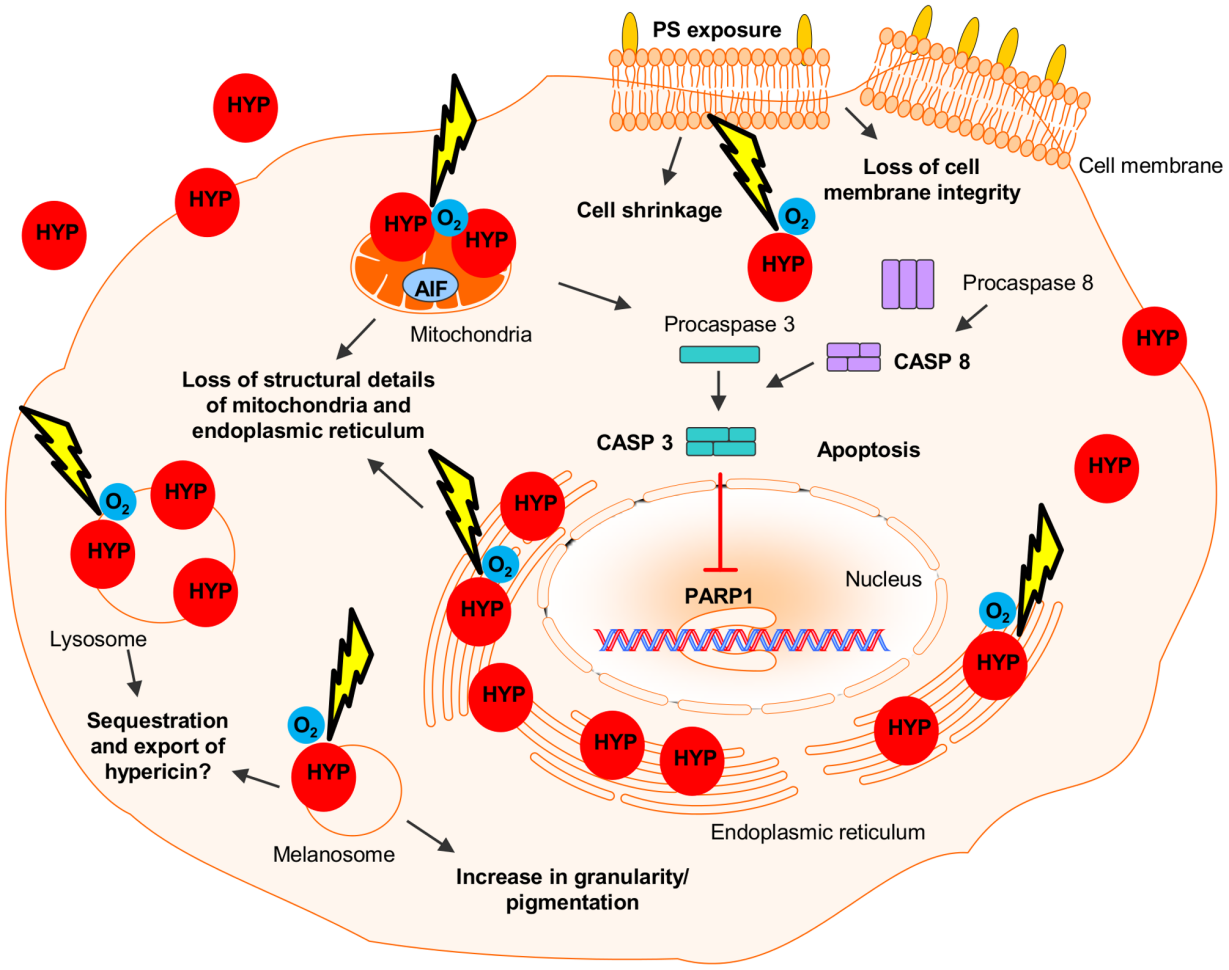
Melanoma response mechanisms to hypericin-PDT. Hypericin (HYP) was taken up by melanoma cells and localized to various intracellular organelles, including the endoplasmic reticulum, mitochondria, lysosomes and melanosomes. Light activation (yellow arrow) of hypericin, in the presence of oxygen (O_2_), resulted in loss of structural details of various intracellular organelles, phosphatidylserine (PS) exposure, loss of melanoma cell membrane integrity, cell shrinkage and an increase in granularity/pigmentation. Hypericin-PDT furthermore initiated caspase-dependent apoptotic modes of cell death of both extrinsic (caspase 8 (CASP8)) and intrinsic (caspase 3 (CASP3)) nature, as well as a caspase-independent apoptotic mode that did not involve apoptosis inducing factor (AIF). Both caspase-dependent and caspase-independent apoptotic modes of cell death resulted in the cleavage of poly(ADP-ribose)polymerase 1 (PARP1).

Overall, this study shows that hypericin was sufficiently taken up by melanoma cells and localized to various intracellular organelles, including the endoplasmic reticulum, mitochondria, lysosomes and melanosomes. Light activation of 3 µM hypericin resulted in photodestruction of mitochondria and the endoplasmic reticulum, phosphatidylserine (PS) exposure, loss of melanoma cell membrane integrity, cell shrinkage and apoptosis (Fig. 8). Mechanistically, the mode of cell death in these cells suggests an initial necrotic, followed by an apoptotic response. As these cellular responses are not absolutely clear-cut, it may suggest an alternative mechanism of necroptosis. Finally, the implications of the increased melanoma cell granularity/pigmentation post hypericin-PDT and the possible use of lysosomal related organelles to sequester and export hypericin from the cell to counteract PDT, remains an interesting avenue to explore for increased therapeutic efficiency against this retractable cancer.

## Supporting Information

Figure S1 **Intracellular localization of hypericin.** Cells were exposed to 3 µM hypericin for 4 h without light activation. Live confocal fluorescent microscopy images of melanoma cells indicate the intracellular localization of hypericin (red) in relation to the endoplasmic reticulum (Bcl2-Cb5-GFP), endosomes, early melanosomes (Rab7-GFP) and mature melanosomes (Rab27a-GFP). Nuclei were counterstained with Hoechst (blue). Profiles taken at different locations through the cell indicate co-localization of the fluorophores. A representative result is shown (n = 3, scale bars: 10/20 µm).
(TIF)

Figure S2 **Phosphatidylserine exposure and loss of cell membrane integrity is not observed in untreated melanoma cells.** Control treatments of hypericin-treated, sham-irradiated (Hypericin −Light), vehicle-treated, irradiated (Vehicle +Light) and vehicle-treated, sham-irradiated (Vehicle −Light) melanoma cells at 30 min, 1, 4, 7 and 24 h after treatment. Data is shown as percentage gated cells of 4 different populations labeled with Annexin V (phosphatidyl serine exposure) and VIVD (loss of cell membrane integrity): AV− VIVD− (live), AV+ VIVID− (early apoptotic), AV− VIVID+ (necrotic) and AV+ VIVID+ (late apoptotic/necrotic). Data is shown as mean±SEM of gated cells (n≥3).
(TIF)

Figure S3 **Hypericin-PDT induced expression of apoptotic proteins.** Caspase 3 (CASP3), caspase 8 (CASP8), poly(ADP-ribose)polymerase 1 (PARP1) and apoptosis inducing factor (AIF) Western blot analyses of whole cell lysates detected at 1, 4, 7 and 24 h after treatment. A representative result of X-ray films of the same exposure is shown (n = 3, CTRL: vehicle-treated control, HYP: hypericin, +C: positive control (doxorubicin-treated), U: untreated, non-irradiated, L: light (-  =  sham-irradiated)).
(TIF)

Video S1 **Hypericin-PDT induced loss of structural details of OTC-GFP positive structures (mitochondria).** Cells expressing OTC-GFP (green) were exposed to 3 µM hypericin (red) for 4 h followed by light-activation with live confocal fluorescent time-lapse microscopy. A cellular region (red box) was bleached with the 561 nm excitation wavelength to activate hypericin. Nuclei were counterstained with Hoechst (blue). A representative time-lapse result is shown (n = 3, scale bars: 20 µm).
(AVI)

Video S2 **Structural details of OTC-GFP positive structures (mitochondria) are not lost in untreated cells.** Control cells expressing OTC-GFP (green) were exposed to vehicle for 4 h followed by light-activation with live confocal fluorescent time-lapse microscopy. A cellular region (red box) was bleached with the 561 nm excitation wavelength to activate hypericin. Nuclei were counterstained with Hoechst (blue). A representative time-lapse result is shown (n = 3, scale bars: 20 µm).
(AVI)
