# Supplementary figures and images for: St John's Wort (Hypericum perforatum L.) Photomedicine: Hypericin-Photodynamic Therapy Induces Metastatic Melanoma Cell Death
Source: PLoS One. 2014 Jul 30;9(7):e103762. doi: 10.1371/journal.pone.0103762 (PMC4116257; doi:10.1371/journal.pone.0103762)

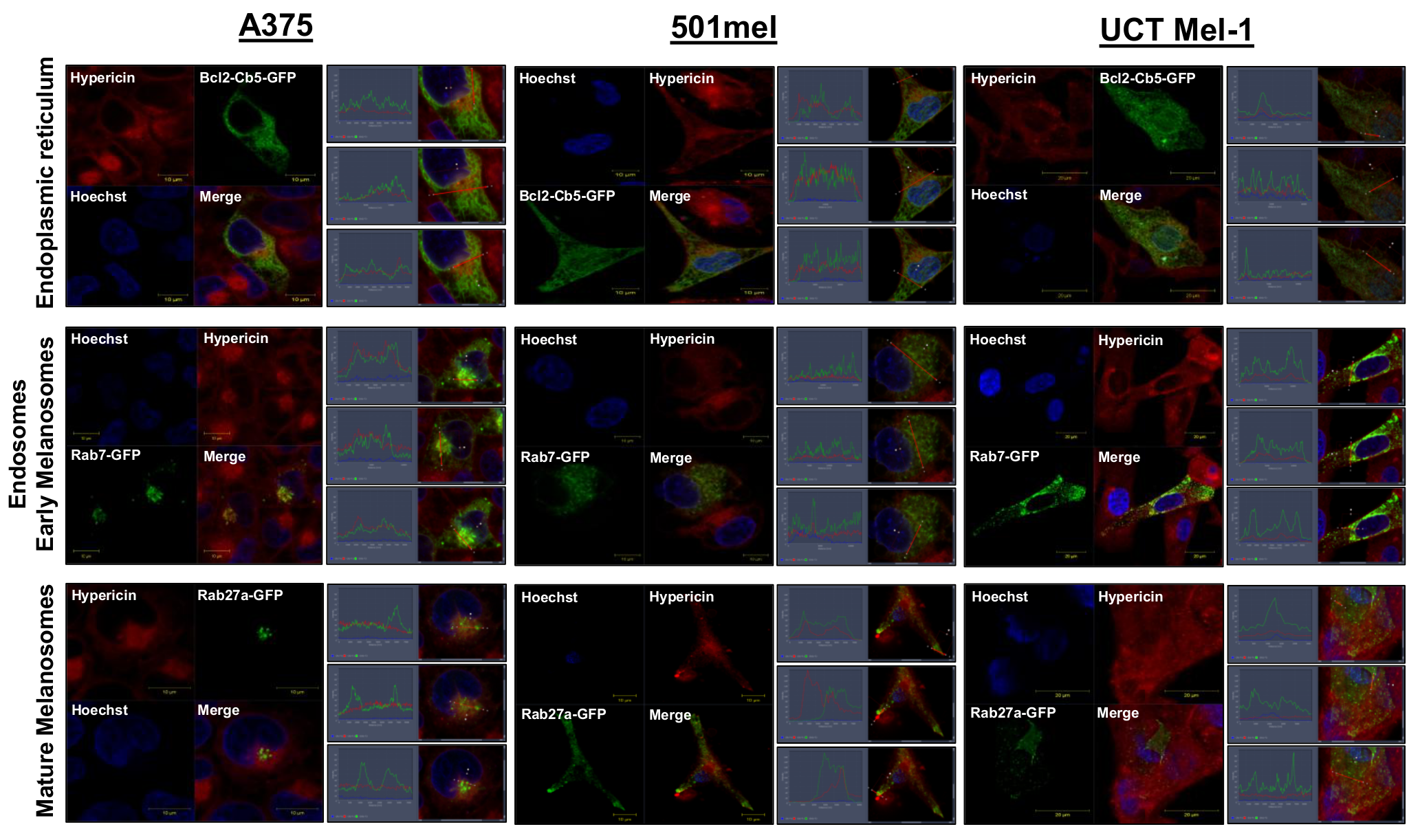

Supplement: Figure S1 — Intracellular localization of hypericin. Cells were exposed to 3 µM hypericin for 4 h without light activation. Live confocal fluorescent microscopy images of melanoma cells indicate the intracellular localization of hypericin (red) in relation to the endoplasmic reticulum (Bcl2-Cb5-GFP), endosomes, early melanosomes (Rab7-GFP) and mature melanosomes (Rab27a-GFP). Nuclei were counterstained with Hoechst (blue). Profiles taken at different locations through the cell indicate co-localization of the fluorophores. A representative result is shown (n = 3, scale bars: 10/20 µm). (TIF) [file pone.0103762.s001.tif]

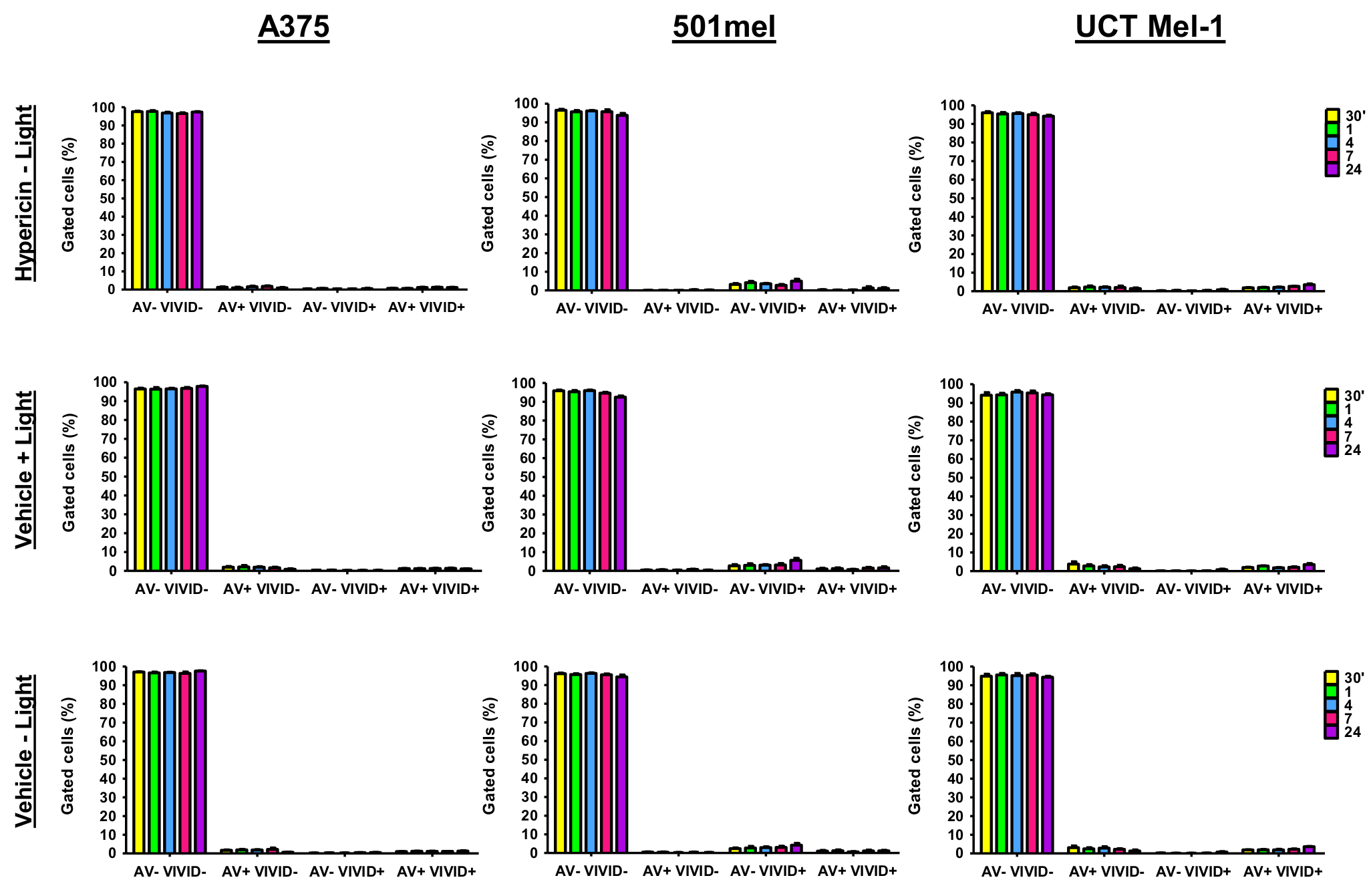

Supplement: Figure S2 — Phosphatidylserine exposure and loss of cell membrane integrity is not observed in untreated melanoma cells. Control treatments of hypericin-treated, sham-irradiated (Hypericin −Light), vehicle-treated, irradiated (Vehicle +Light) and vehicle-treated, sham-irradiated (Vehicle −Light) melanoma cells at 30 min, 1, 4, 7 and 24 h after treatment. Data is shown as percentage gated cells of 4 different populations labeled with Annexin V (phosphatidyl serine exposure) and VIVD (loss of cell membrane integrity): AV− VIVD− (live), AV+ VIVID− (early apoptotic), AV− VIVID+ (necrotic) and AV+ VIVID+ (late apoptotic/necrotic). Data is shown as mean±SEM of gated cells (n≥3). (TIF) [file pone.0103762.s002.tif]
